# Combining Phylogenetic and Network Approaches to Identify HIV-1 Transmission Links in San Mateo County, California

**DOI:** 10.3389/fmicb.2018.02799

**Published:** 2018-12-06

**Authors:** Sudeb C. Dalai, Dennis Maletich Junqueira, Eduan Wilkinson, Renee Mehra, Sergei L. Kosakovsky Pond, Vivian Levy, Dennis Israelski, Tulio de Oliveira, David Katzenstein

**Affiliations:** ^1^Division of Infectious Diseases and Geographic Medicine, Department of Medicine, Stanford University School of Medicine, Stanford, CA, United States; ^2^Division of Epidemiology, School of Public Health, University of California, Berkeley, Berkeley, CA, United States; ^3^KwaZulu-Natal Research Innovation and Sequencing Platform, Nelson R Mandela School of Medicine, University of KwaZulu-Natal, Durban, South Africa; ^4^School of Laboratory Medicine and Medical Science, Department of Health Sciences, University of KwaZulu-Natal, Durban, South Africa; ^5^Division of Hematology, Stanford University School of Medicine, Stanford, CA, United States; ^6^Institute for Genomics and Evolutionary Medicine, Temple University, Philadelphia, PA, United States; ^7^San Mateo Medical Center, San Mateo, CA, United States; ^8^Department of Global Health, University of Washington, Seattle, WA, United States

**Keywords:** HIV, transmission links, California, phylogenetics, network

## Abstract

The HIV epidemic in San Mateo County is sustained by multiple overlapping risk groups and is an important hub for HIV transmission in northern California. Limited access to care has led historically to delayed clinical presentation, higher rates of opportunistic infections, and an increased prevalence of antiretroviral drug resistance. The virologic and clinical consequences of treatment within these multiple ethnic and behavioral groups are poorly understood, highlighting the need for efficient surveillance strategies that are able to elucidate transmission networks and drug resistance patterns. We obtained sequence data from a group of 316 HIV-positive individuals in the San Mateo AIDS Program over a 14-year period and integrated epidemiologic, phylogenetic, and network approaches to characterize transmission clusters, risk factors and drug resistance. Drug resistance mutations were identified using the Stanford HIV Drug Resistance Database. A maximum likelihood tree was inferred in RAxML and subjected to clustering analysis in Cluster Picker. Network analysis using pairwise genetic distances was performed in HIV-TRACE. Participants were primarily male (60%), white Hispanics and non-Hispanics (32%) and African American (20.6%). The most frequent behavior risk factor was male-male sex (33.5%), followed by heterosexual (23.4%) and injection drug use (9.5%). Nearly all sequences were subtype B (96%) with subtypes A, C, and CRF01_AE also observed. Sequences from 65% of participants had at least one drug resistance mutation. Clustered transmissions included a higher number of women when compared to non-clustered individuals and were more likely to include heterosexual or people who inject drugs (PWID). Detailed analysis of the largest network (*N* = 47) suggested that PWID played a central role in overall transmission of HIV-1 as well as bridging men who have sex with men (MSM) transmission with heterosexual/PWID among primarily African American men. Combined phylogenetic and network analysis of HIV sequence data identified several overlapping risk factors in the epidemic, including MSM, heterosexual and PWID transmission with a disproportionate impact on African Americans and a high prevalence of drug resistance.

## Introduction

Molecular epidemiologic analyses of the HIV-1 epidemics in Africa (Dalai et al., 2009; Gray et al., 2009; Mir et al., 2016), Asia (Neogi et al., 2012; Pang et al., 2012), and North America (Smith et al., 2010; Mehta et al., 2015) have provided evidence for distinct epidemic dynamics and patterns of transmission within defined communities. While the southern African and Asian epidemics are thought to be sustained by sexual-social factors, high-risk people who inject drugs (PWID), and commercial sex work (CSW) (Thorne et al., 2010; Baral et al., 2012; de Oliveira et al., 2017), male-male sex has remained the primary mode of transmission in the United States, accounting for approximately 70% of new infections (Centers for Disease Control and Prevention, 2015). In the past 10 years, the HIV epidemic in California has shifted from a primarily white MSM (men who have sex with men) population to include a diverse range of overlapping risk groups, where heterosexual women comprise the fastest growing demographic for new infections and an increasing fraction of new diagnoses occur among diverse racial and ethnic groups (California Department of Public Health, 2015). Hispanic monolingual men and women now predominate among new HIV-1 cases in several California counties followed by African Americans and Asian/Pacific Islanders (Magis-Rodríguez et al., 2009).

The HIV epidemic in San Mateo County (California) encompasses approximately 1,613 diagnosed cases with 0.2% adult prevalence^1^. The epidemic is sustained by multiple ethnic, migratory, and behavioral networks, including MSM, migrant populations from Asia and Latin America, and PWID, each having distinct patterns of HIV acquisition and transmission (San Mateo County Department of Public Health, 2017). Within these communities, access to care, including antiretroviral treatment (ART) has historically been constrained by financial, cultural, and linguistic barriers. HIV-positive immigrants (78.7% Hispanic) attending the publicly-funded San Mateo County AIDS Program have substantially delayed clinical presentation, marked by lower baseline CD4+ cell counts, greater prevalence of opportunistic infections, and higher hospitalization rates as compared with United States-born individuals (Levy et al., 2006). Moreover, population-based surveys in San Mateo have recently identified multiple risk behaviors associated with immigrant status, including unprotected sex, unstable and overlapping sexual partnerships, CSW contacts, and PWID (Levy et al., 2005). The implications of prolonged, undiagnosed HIV infection, viremia, delayed treatment, and expanded risk behavior for community transmission networks remain largely uncharacterized.

In California, a high prevalence of drug resistance among untreated, newly diagnosed patients has highlighted widespread community-level transmission and cross-border introduction of multi-drug resistant HIV associated with an expansion in migratory and risk behaviors (Panichsillapakit et al., 2016). In recent years a focus on addressing health disparities in California has increased access to treatment and care for historically marginalized populations. However, data are limited regarding the virologic and clinical outcomes of treatment in these communities. These observations underscore the need for broad and systematic surveillance of HIV transmission networks and drug resistance in the context of comprehensive health service delivery systems, particularly among populations who present late and where ART treatment access is constrained.

Defining the patterns of HIV transmission and resistance within communities is important for regional prevention and treatment programs to develop effective, integrated testing and treatment strategies to reduce transmission and to identify and appropriately treat newly-infected individuals. This study combines phylogenetic inference, network analysis and molecular virology/epidemiology to characterize HIV viral transmission and ARV drug resistance in a northern California community epidemic over a 14-year period.

## Materials and Methods

### Study Population

The study population included 316 HIV-positive adults receiving care as part of the publicly funded San Mateo County AIDS Program who underwent clinically indicated genotypic antiretroviral resistance testing (GART) from 1996 to 2010. The population included both acutely- and chronically-infected, as well as treatment naïve and multi-drug experienced individuals. Demographic (age, gender, race/ethnicity), epidemiologic (date of diagnosis, mode of transmission, social/risk behavior, partner information, location data), and clinical information (history of ART usage, HIV clinical stage, HIV viral load, CD4+ cell count, co-infections) were de-identified and extracted from electronic and written medical records at San Mateo Medical Center. Mode of HIV transmission was extracted from medical records as determined by the physician at the time of patient intake. Transmission categories included (1) MSM; (2) PWID; (3) MSM + PWID; and (4) heterosexual/other (including participants reporting infection through contaminated blood products). Missing values were treated as a separate category for all demographic variables analyzed in this study.

The use of anonymized, de-identified clinical/demographic and sequence data was reviewed and approved under an exempt protocol by the Institutional Review Boards of Stanford University, the University of California, Berkeley, and Mills-Peninsula Health Services on behalf of San Mateo Medical Center. All subjects gave written informed consent in accordance with the Declaration of Helsinki.

### Sequence Data and Alignment

Genotypic resistance assays were performed as part of standard clinical care by the Stanford Hospital Clinical Virology Laboratory. A total of 637 HIV-1 *pol* gene sequences were obtained from 316 patients tested over the study period. Sequences were generated by dideoxynucleotide sequencing of population (consensus) amplicons from HIV-1 *pol*. Sequences were aligned and manually edited using the ClustalW algorithm as implemented in BioEdit (Hall, 1999). For individuals who had multiple sequences, the earliest available sequence was retained for transmission/clustering analyses as well as for the screening of drug resistance mutations.

Additionally, a reference sequence dataset was compiled using BLAST+ (Camacho et al., 2009). For each study sequence, the 50 most similar reference sequences from BLAST were selected, resulting in a reference alignment of 1,405 sequences after removal of duplicates.

### Subtype Classification and Drug Resistance Analysis

HIV-1 subtype and evidence for inter-subtype recombination were assessed using the REGA Subtyping Tool v3.0 (de Oliveira et al., 2005; Alcantara et al., 2009). The HIVseq algorithm was used to interpret genotypic resistance and to identify known ART drug resistance mutations (DRMs) according to the most recent International AIDS Society (IAS) mutation list (Wensing et al., 2017). Both methods were implemented in the Stanford HIV Drug Resistance Database (Gifford et al., 2009).

### Phylogenetic and Clustering Analysis

A maximum likelihood (ML) phylogenetic tree was constructed in RAxML (Stamatakis, 2014) using the general time reversible model of nucleotide substitution (Tavaré, 1986), an estimated proportion of invariant sites and a gamma correction for among-site rate variation. Statistical support for internal nodes was obtained via bootstrapping with 1000 replicates. Alternatively, branch supports were also calculated via transfer bootstrap expectation (TBE) in BOOSTER (Lemoine et al., 2018) using all 1000 replicates generated in RAxML. TBE provides optimized support for deep branches in large phylogenetic trees when compared to the classical bootstrap criterion, allowing for the identification of large putative clusters. Trees were visualized using FigTree v1.4.3 (Rambaut, 2009).

In order to assess potential bias from DRMs, comparative phylogenetic trees were constructed in PhyML (Guindon et al., 2005) from a separate sequence dataset including only samples from San Mateo. All IAS codons associated with major antiretroviral drug resistance (Wensing et al., 2017) were excluded from one of these alignments. Reliability of the obtained topologies was estimated with 1000 bootstrap replicates. Notably, inclusion or removal of DRMs did not appreciably alter tree structure.

Transmission clusters were identified with the program Cluster Picker (Ragonnet-Cronin et al., 2013) using a minimum branch support of 90 (bootstrap or BTE) and an intra-cluster genetic distance threshold of 4 percent or 8 percent. Clusters including more than 5 individuals were separately evaluated in TempEst to investigate their temporal signal. When a molecular clock assumption was validated, these clusters were submitted for Bayesian phylogenetic reconstruction using BEAST v1.8.4 (Rambaut et al., 2016; Suchard et al., 2018). In addition to a phylogenetic approach, HIV-TRACE was used to reconstruct a putative genetic transmission network with a pairwise genetic distance of 2%, which is in the 1 – 2% range derived from several comparative studies of epidemiologically-linked partners and studies of within-host evolutionary rates (Wertheim et al., 2014). HIV-TRACE performs agglomerative hierarchical clustering connecting sequences only if their pairwise distance does not exceed the assigned threshold. As an analog to phylogenetic bootstrap, i.e., to assess network sensitivity to sampling error, we repeated network inference on 100 bootstrap replicates created from the original alignment with goalign^2^. Medical records were independently reviewed to identify epidemiologic linkages among study participants irrespective of the phylogenetic/network linkages. Univariate and multivariate statistical analyses of risk factors associated with clustering were performed in the statistical package R (R Core Team, 2013).

## Results

### Patient Demographics

Between 1996 and 2010, 637 HIV sequences were obtained from 316 people living with HIV/AIDS (PLWHA) receiving ART in the San Mateo County AIDS Program. Table 1 summarizes baseline demographic characteristics of the participants. The study population was comprised primarily of white Hispanics and non-Hispanics (32.0%) and African Americans (20.6%). Around 60% of participants were male, with ages ranging from 19 to 67 years old. The most frequent recorded mode of HIV transmission was MSM (33.5%), followed by heterosexual (23.4%), PWID (9.5%), and combined MSM/PWID (8.5%). Three participants reported infection through contaminated blood products.

**Table 1:** Demographic and epidemiological data for 316 HIV-positive adults receiving antiretroviral treatment in San Mateo County, California.

	Clustered Sequences		Total (*n*=316)
	Cluster Picker (*n*=66)	HIV-TRACE (*n*=61)	
Gender			
Male	32 (48.48%)	31 (50.82%)	190 (60.13%)
Female	25 (37.88%)^∗^	18 (29.51%)^∗^	49 (15.5%)
Transgender/Unknown	9 (13.64%)	12 (19.67%)	77 (24.37%)
Age (years)	45.2 (1.1)	43.8 (1.2)	41.7 (0.5)
**Primary Language**			
English	47 (71.21%)	39 (63.93%)	185 (58.5%)
Spanish	5 (7.58%)	4 (6.56%)	26 (8.2%)
Bilingual	3 (4.55%)	2 (3.28%)	12 (3.8%)
Other/Unknown	11 (16.67%)	16 (26.23%)	93 (29.4%)
**Ethnicity**			
Black	39 (59.09%)^∗^	23 (37.7%)^∗^	65 (20.6%)
White	7 (10.61%)	14 (22.95%)	101 (32.0%)
Asian	1 (1.52%)	4 (6.56%)	13 (4.1%)
Latino	8 (12.12%)	6 (9.84%)	46 (14.6%)
Other/Unknown	11 (16.67%)	14 (22.95%)	91 (28.8%)
**Hispanic ancestry**			
No	48 (72.73%)^∗^	40 (65.57%)	187 (59.2%)
Yes	9 (13.64%)	9 (14.75%)	53 (16.8%)
Unknown	9 (13.64%)	12 (19.67%)	76 (24.1%)
**Mode of transmission**			
Heterosexual	29 (43.94%)^∗^	23 (37.7%)^∗^	74 (23.4%)
PWID	19 (28.79%)^∗^	12 (19.67%)^∗^	30 (9.5%)
MSM	4 (6.06%)	11 (18.03%)	106 (33.5%)
MSM/PWID	5 (7.58%)	2 (3.28%)	27 (8.5%)
Unknown	9 (13.64%)	13 (21.31%)	79 (25.0%)
**Drug Resistance Mutation**			
PI	17 (25.76%)	8 (13.11%)	87 (27.5%)
NRTI	38 (57.58%)	22 (36.07%)	186 (58.9%)
NNRTI	11 (16.67%)	4 (6.56%)	74 (23.4%)

*Data are n (%) or mean (SE). MSM, men who have sex with men; PWID, people who inject drugs, injection drug users; PI, protease inhibitor; NRTI, nucleoside reverse transcriptase inhibitors; NNRTI, non- nucleoside reverse transcriptase inhibitors. ^∗^p < 0.05 (cluster vs. non-clustered comparisons for each method).*

### Transmission Clustering

**FIGURE 1:**
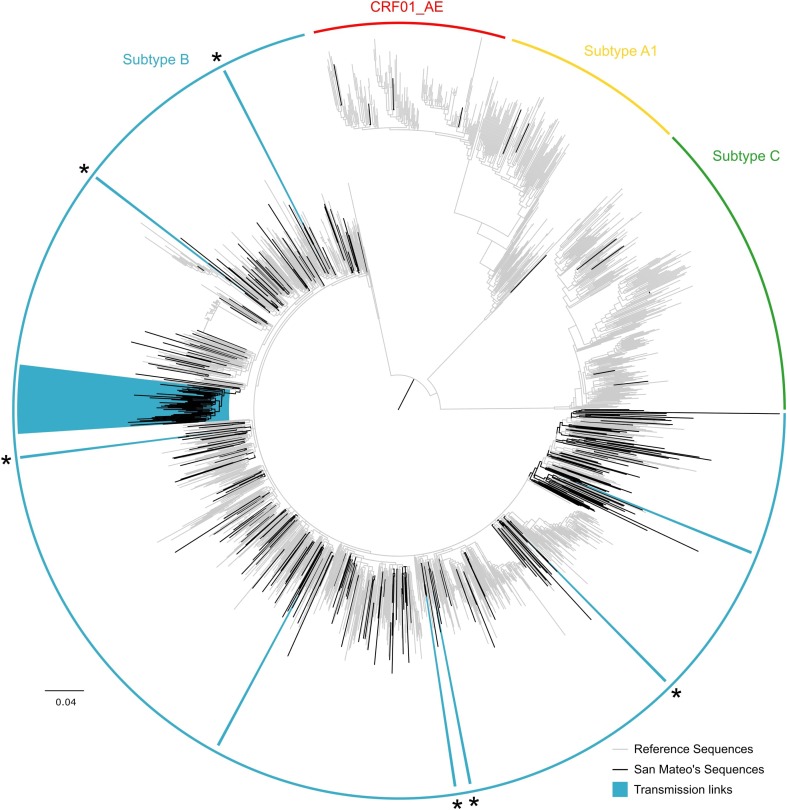
Maximum likelihood HIV-1 *pol* transmission clusters in San Mateo County, California. Colored semi-circles indicate HIV subtypes. Black branches represent sequences obtained from 316 HIV-positive adults receiving antiretroviral treatment as part of the publicly funded San Mateo County AIDS Program. Gray branches denote reference sequences. Clades highlighted in blue indicate transmission clusters identified in Cluster Picker (TBE ≥ 90 and genetic distance threshold of 8%). Asterisks indicate transmission pairs mutually identified as transmission links in Cluster Picker and HIV-TRACE. The scale bar at the bottom represents the number of substitutions per site along branches in the tree topology.

Of the 316 genotypes, nearly all sequences were subtype B (95.9%, Figure 1). Six sequences (1.9%) were subtype C, four individuals (1.3%) were infected with the circulating recombinant form CRF01_AE, and three sequences (0.9%) were subtype A.

**FIGURE 2:**
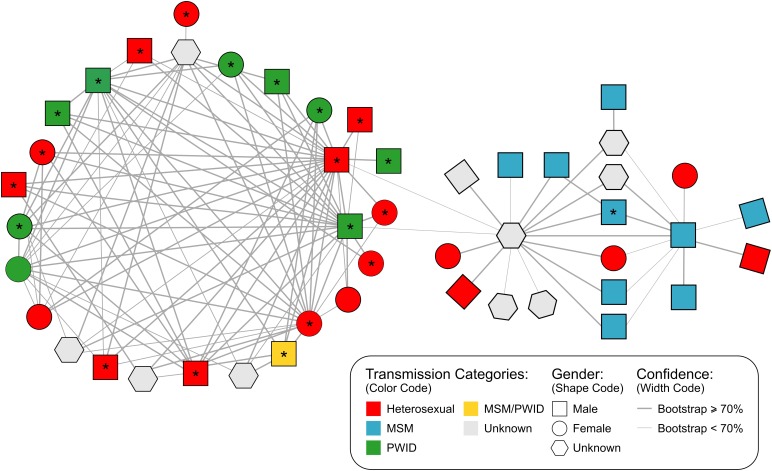
Inferred genetic network structure of a major cluster (N = 47 individuals) in the San Mateo HIV-1 subtype B epidemic identified by HIV-TRACE using a genetic distance threshold of 2%. Shape codes gender, colors denote the reported mode of transmission for each individual and line width represents the bootstrap confidence for linkage. ^∗^are indicating African-American individuals. MSM, men who have sex with men; PWID, people who inject drugs, injection drug users.

Sequences from San Mateo were combined with 1,405 reference sequences identified as the most similar sequences from GenBank. Cluster Picker identified 8 and 9 phylogenetic clusters (TBE bootstrap > 90%) at intra-cluster genetic thresholds of 4 and 8%, respectively. At the 4% intra cluster genetic distance threshold, the phylogenetic tree included seven transmission pairs and one putative cluster of three individuals. At an 8% cut-off, eight transmission pairs and one large single cluster (*n* = 50) were identified. No significant correlation between sampling date and evolutionary rate was found for this large cluster in TempEst (slope: -0.0013, R2: 0.093443), which implies that phylodynamic characterization would not be indicated. Nevertheless, sequences were submitted to a Bayesian analysis and after 4 × 10^8^ million steps, runs did not converge (ESS > 13) and precluded further estimates. Network analysis with HIV-TRACE detected seven transmission pairs and a single putative transmission network of 47 individuals (Figure 2). Five of the seven transmission pairs were also identified in the Cluster Picker analysis (Supplementary Table S1).

Cluster Picker and HIV-TRACE identified clusters having a similar demographic composition (Table 1). In a univariate analyses, clustered individuals included a higher proportion of women when compared to non-clustered patients (*p* < 0.001). In addition, clusters were more likely to include heterosexual or PWID (*p* < 0.001) mode of transmission and African Americans (*p* < 0.01). Notably, individuals reporting MSM mode of transmission were less likely to be in clusters (*p* < 0.001). No association was seen between clustering and age or presence of DRMs (PI, NRTI or NNRTI).

Further analysis of the large network identified by HIV-TRACE suggested that PWID and MSM played a central role in the transmission of HIV-1 in this putative transmission network. Based on bootstrap support the network is divided into two different sub-clusters (Figure 2): (i) the larger subgroup includes 27 PWID or heterosexual individuals that are linked with a high, well-supported degree of connectedness (2.7 links per person supported by a bootstrap > 70%) and has a overall genetic diversity of 0.022 [bootstrap procedure standard error (SE): 0.002062], (ii) a second cluster bridging to the first by two weakly supported links (63 and 65% bootstraps) is comprised mainly of MSM individuals (*n* = 9) followed by heterosexual (*n* = 5) and unknown risk group individuals (*n* = 6) and has an intra-genetic diversity of 0.027 (SE: 0.002315). The genetic distance between sub-groups was 0.033 (SE: 0.003280).

### Drug Resistance

Of 305 participants with available drug resistance data, 65% had at least one DRM. The most frequent nucleoside reverse transcriptase inhibitor (NRTI) resistance mutations were M184V (49.5%), T215Y (26.9%), and M41L (20.3%). The most frequent non-nucleoside reverse transcriptase inhibitor (NNRTI) mutations were K103N (15.4%), G190E (3%), and K101E (2.6%). The most frequent protease inhibitor (PI) mutations were at positions 46 (8.9%), 54 (7.2%) and 82 (26%). Factors associated with drug resistance included: male gender; chronic HIV infection; diagnosis with HIV and genotyping performed during an earlier (as opposed to more recent) time period; lower viral load (3.9 vs. 4.4 log_10_ copies/mL, *p* = 0.006); and NRTI or NNRTI treatment. After multivariate adjustment, factors independently associated with drug resistance of any class included being male, genotyping during an earlier time period, lower viral load, and exposure to NNRTIs.

## Discussion

Phylogenetic analyses have been widely used to define and characterize transmission links among HIV-infected individuals (Baral et al., 2012; Wertheim et al., 2014; Rambaut et al., 2016; de Oliveira et al., 2017; Kostaki et al., 2018). San Mateo County has a unique and heterogeneous population of individuals living with HIV/AIDS with distinct ethnic, racial and language communities overlapping the traditional behavioral risk-groups of MSM, heterosexual and PWID. Relative to county demographics, African American individuals are over-represented and Asian Americans underrepresented among patients presenting for evaluation and treatment in the San Mateo AIDS Program (San Mateo County Sexually Transmitted Disease and HIV-AIDS Surveillance Annual Report, 2015).

Combined phylogenetic and network analyses among HIV-positive adults from San Mateo County have identified a striking role of intra-community transmission dynamics among African Americans and highlight the importance of epidemiological bridging between risk groups in the local epidemic. As expected, we found some differences in the number and composition of clusters detected using two different methods (Rose et al., 2017), which may be attributed to different underlying methodologies. HIV-TRACE uses pairwise genetic distances between isolates to build putative transmission networks, while Cluster Picker relies on the intra-cluster genetic distance within putative clusters in a phylogenetic tree in conjunction with branch support. By using a minimum branch support of 90% (TBE) and an intra-cluster genetic distance threshold of 8%, we identified nine supported clusters in Cluster Picker. In contrast, with a maximum pairwise genetic distance of 2%, the transmission network approach in HIV-TRACE detected eight clusters (Supplementary Table S1). Transmission pairs had a concordance of 67% between the two methods. Interestingly, both methods identified one large cluster of linked individuals (Figures 1, 2). In addition, the clusters commonly identified had similar demographic composition.

Network analysis of the large 47-person cluster using HIV-TRACE suggests the existence of a highly-connected linked chain of transmission comprising individuals of diverse demographic backgrounds. This network included two sub-clusters with different primary modes of HIV transmission (Figure 2). Twenty-seven individuals were substantially interconnected in a heterosexual and/or PWID transmission chain mainly involving African American individuals. Connected to this cluster is a less-intricate network primarily composed of white MSM. The linkage between the two sub-clusters included two males (PWID and heterosexual) and an individual of unknown gender but highly connected to MSM. In such a scenario, some individuals may provide a bridge between the MSM and heterosexual epidemics mostly likely through PWID. Alternatively, this may reflect the well-documented epidemiological phenomena of bisexual black males who do not identify as gay and do not disclose high-risk MSM behavior to their female partners, but subsequently contribute disproportionately to heterosexual HIV transmission (Millett et al., 2005; Bond et al., 2009). The fact that the inter-genetic distance between sub-clusters is higher than that within groups may explain the weak association (63 and 65% bootstraps) between the two sub-clusters and may potentially reflect the result of a remote transmission link.

Risk factors associated with transmission clustering include heterosexual or PWID transmission modes, female gender and African descent. MSM was not associated with clustering in this sample. These results are consistent with epidemiologic observations in San Mateo County in San Mateo County where a diverse range of overlapping risk groups, particularly heterosexual women, is identified among new infections (Levy et al., 2006). A previous study of 96 large United States metropolitan areas demonstrated higher AIDS incidence and mortality among heterosexuals in areas with a higher population prevalence of HIV-positive PWID as well as decreased presence of needle-exchange or drug-use treatment programs (Friedman et al., 2014). These findings corroborate our results and suggest that the burden of HIV/AIDS among heterosexuals in more recent years may have been shaped by bridging from HIV-infected PWID to heterosexuals and possibly, though to a lesser degree, from HIV-positive MSM to heterosexuals. These results highlight that efforts to mitigate transmission among PWID, including HIV counseling and testing or drug use treatment, can directly impact transmission to other key populations (World Health Organization, 2012; Friedman et al., 2014). Despite the predominance of Hispanic monolingual men and women among new HIV-cases in California (Levy et al., 2005), no clusters in this study had a significant representation of Spanish-speaking individuals. However, a substantial number of individuals included in clusters were diagnosed before 1999, suggesting that our sampling and methods identified remote transmission links that may not necessarily reflect current epidemic dynamics.

This study has some limitations and potential biases. The demographic composition of patients in the San Mateo AIDS Program differs substantially from the overall population in San Mateo County; notably, Black or African Americans accounted for 20.6% of the study population while comprising only 3% of the county population annually, and Asians accounted for only 4.1% of the study population while annually they comprise 24–29% of the county demographic. Patients attending the San Mateo AIDS Program also have lower average income than the county median (Levy et al., 2006), highlighting the health inequalities underlying transmission of HIV and other sexually transmitted infections.

Finally, the sampling frame defined within San Mateo County is restrictive. Several study subjects had previously moved from neighboring areas to San Mateo to access treatment, whereas others indicated they were infected or diagnosed in other US states or outside the United States. In comparable community-based studies comprised of predominantly MSM, large transmission clusters have been identified (Smith et al., 2010; Mehta et al., 2015). The lack of large clustering in the present study despite nearly 33% MSM participants suggests the existence of uncaptured, overlapping transmission networks in other geographic locations. For example, the lack of clustering among monolingual White Hispanics may reflect transitory migration and infection with HIV strains that originated along the Mexico-California border, or, alternatively, a bias toward selecting older strains that were circulating earlier in the epidemic, as immigrant status has been independently associated with delayed presentation, diagnosis and care (Sanchez et al., 2004; Levy et al., 2005, 2006; Dennis et al., 2015). Finally, there was incomplete availability of epidemiological and clinical data largely due to destruction or remote storage of archived medical records, limiting the statistical power of clustering and drug resistance analyses.

## Conclusion

As HIV genotypic data become routinely available for molecular epidemiologic analyses, initial treatment options and public health approaches to ART implementation can be optimized to avoid early virologic failure particularly in the setting of resource limitations. The high frequency of drug resistance (65.5%) identified in this population most likely reflects a substantial burden of long-standing HIV disease with heavy ART exposure (89.2%). This highlights the importance of genotypic testing to determine the most effective ARV regimen for newly-diagnosed individuals. Our finding of over 4% non-subtype B HIV in the San Mateo AIDS Program (largely among immigrants and transitory individuals), including subtypes A, C, and recombinant CRF01_AE, provides evidence for the introduction of HIV variants other than subtype B into the community. Integration of phylogenetic and network methodologies to identify putative transmission links detected a large cluster with demographic and risk group composition reflecting the HIV epidemic in San Mateo County. Female gender, African-descent and heterosexual intercourse or PWID seem to be key features of these transmission networks. Despite aggressive epidemiologic surveillance, HIV awareness campaigns, and behavior change programs, the rate of new HIV infections has increased or remained constant in many United States communities. High-resolution molecular epidemiologic analyses are useful means of identifying sources of viral diversity and excess transmission risk and, when integrated with broad surveillance, can contribute to strategies to reduce new HIV infections.

## Data Availability

GenBank accession numbers of the sequences described in this study: MK025232 – MK025548.

## Author Contributions

SD, TdO, and DK conceived and designed the research. SD, DJ, EW, RM, VL, and SLKP conducted the research. SD, RM, VL, and DI involved in the patient data collection and clinical care. SD, RM, and VL performed the sample collection and molecular analysis. SD, DJ, EW, and SLKP performed the data analysis and created graphics. SD and DJ wrote the manuscript, with the assistance of all co-authors.

## Conflict of Interest Statement

The authors declare that the research was conducted in the absence of any commercial or financial relationships that could be construed as a potential conflict of interest.

## Footnotes

1 http://www.countyhealthrankings.org/

2 https://github.com/fredericlemoine/goalign

## References

[B1] Alcantara L. C. J. Cassol S. Libin P. Deforche K. Pybus O. G. Van Ranst M. (2009). A standardized framework for accurate, high-throughput genotyping of recombinant and non-recombinant viral sequences. *Nucleic Acids Res.* 37 1–9. 10.1093/nar/gkp455 19483099 PMC2703899

[B2] Baral S. Beyrer C. Muessig K. Poteat T. Wirtz A. L. Decker M. R. (2012). Burden of HIV among female sex workers in low-income and middle-income countries: a systematic review and meta-analysis. *Lancet Infect. Dis.* 12 538–549. 10.1016/S1473-3099(12)70066-X 22424777

[B3] Bond L. Wheeler D. P. Millett G. A. LaPollo A. B. Carson L. F. Liau A. (2009). Black men who have sex with men and the association of down-low identity with HIV risk behavior. *Am. J. Public Health* 99(Suppl. 1), 92–95. 10.2105/AJPH.2007.127217 19218177 PMC2724949

[B4] California Department of Public Health (2015). *California HIV Surveillance Report.* Sacramento, CA: California Department of Public Health.

[B5] Camacho C. Coulouris G. Avagyan V. Ma N. Papadopoulos J. Bealer K. (2009). BLAST+: architecture and applications. *BMC Bioinformatics* 10:421. 10.1186/1471-2105-10-421 20003500 PMC2803857

[B6] Centers for Disease Control and Prevention (2015). *HIV Surveillance Report.* Atlanta, GA: Centers for Disease Control and Prevention.

[B7] Dalai S. C. de Oliveira T. Harkins G. W. Kassaye S. G. Lint J. Manasa J. (2009). Evolution and molecular epidemiology of subtype C HIV-1 in Zimbabwe. *AIDS* 23 2523–2532. 10.1097/QAD.0b013e3283320ef3 19770693 PMC2923658

[B8] de Oliveira T. Deforche K. Cassol S. Salminen M. Paraskevis D. Seebregts C. (2005). An automated genotyping system for analysis of HIV-1 and other microbial sequences. *Bioinformatics* 21 3797–3800. 10.1093/bioinformatics/bti607 16076886

[B9] de Oliveira T. Kharsany A. B. M. Gräf T. Cawood C. Khanyile D. Grobler A. (2017). Transmission networks and risk of HIV infection in KwaZulu-Natal, South Africa: a community-wide phylogenetic study. *Lancet HIV* 4 e41–e50. 10.1016/S2352-3018(16)30186-2 27914874 PMC5479933

[B10] Dennis A. M. Hué S. Pasquale D. Napravnik S. Sebastian J. Miller W. C. (2015). HIV transmission patterns among immigrant Latinos illuminated by the integration of phylogenetic and migration data. *AIDS Res. Hum. Retroviruses* 31 973–980. 10.1089/AID.2015.0089 26214548 PMC4576933

[B11] Friedman S. R. West B. S. Tempalski B. Morton C. M. Cleland C. M. Des Jarlais D. C. (2014). Do metropolitan HIV epidemic histories and programs for people who inject drugs and men who have sex with men predict AIDS incidence and mortality among heterosexuals? *Ann. Epidemiol.* 24 304–311. 10.1016/j.annepidem.2014.01.008 24529517 PMC3954755

[B12] Gifford R. J. Liu T. F. Rhee S.-Y. Kiuchi M. Hue S. Pillay D. (2009). The calibrated population resistance tool: standardized genotypic estimation of transmitted HIV-1 drug resistance. *Bioinformatics* 25 1197–1198. 10.1093/bioinformatics/btp134 19304876 PMC2672634

[B13] Gray R. R. Tatem A. J. Lamers S. Hou W. Laeyendecker O. Serwadda D. (2009). Spatial phylodynamics of HIV-1 epidemic emergence in east Africa. *AIDS* 23 F9–F17. 10.1097/QAD.0b013e32832faf61 19644346 PMC2742553

[B14] Guindon S. Lethiec F. Duroux P. Gascuel O. (2005). PHYML Online—a web server for fast maximum likelihood-based phylogenetic inference. *Nucleic Acids Res.* 33 W557–W559. 10.1093/nar/gki352 15980534 PMC1160113

[B15] Hall T. A. (1999). BioEdit: a user-friendly biological sequence alignment editor and analysis program for Windows 95/98/NT. *Nucleic Acids Symp. Ser.* 41 95–98.

[B16] Kostaki E.-G. Nikolopoulos G. K. Pavlitina E. Williams L. Magiorkinis G. Schneider J. (2018). Molecular analysis of human immunodeficiency virus type 1 (HIV-1)-infected individuals in a network-based intervention (transmission reduction intervention project): phylogenetics identify HIV-1-infected individuals with social links. *J. Infect. Dis.* 218 707–715. 10.1093/infdis/jiy239 29697829 PMC6057507

[B17] Lemoine F. Domelevo Entfellner J. B. Wilkinson E. Correia D. Dávila Felipe M. De Oliveira T. (2018). Renewing Felsenstein’s phylogenetic bootstrap in the era of big data. *Nature* 556 452–456. 10.1038/s41586-018-0043-0 29670290 PMC6030568

[B18] Levy V. Page-Shafer K. Evans J. Ruiz J. Morrow S. Reardon J. (2005). HIV-related risk behavior among hispanic immigrant men in a population-based household survey in low-income neighborhoods of northern California. *Sex. Transm. Dis.* 32 487–490. 10.1097/01.olq.0000161185.06387.94 16041250

[B19] Levy V. Prentiss D. Balmas G. Chen S. Israelski D. Katzenstein D. (2006). Factors in the delayed HIV presentation of immigrants in northern California: implications for voluntary counseling and testing programs. *J. Immigr. Minor. Heal.* 9 49–54. 10.1007/s10903-006-9015-9 17031578

[B20] Magis-Rodríguez C. Lemp G. Hernandez M. T. Sanchez M. A. Estrada F. Bravo-García E. (2009). Going North: mexican migrants and their vulnerability to HIV. *JAIDS J. Acquir. Immune Defic. Syndr.* 51 S21–S25. 10.1097/QAI.0b013e3181a26433 19384097

[B21] Mehta S. R. Wertheim J. O. Brouwer K. C. Wagner K. D. Chaillon A. Strathdee S. (2015). HIV transmission networks in the San Diego–Tijuana border region. *EBioMedicine* 2 1456–1463. 10.1016/j.ebiom.2015.07.024 26629540 PMC4634195

[B22] Millett G. Malebranche D. Mason B. Spikes P. (2005). Focusing “down low”: bisexual black men, HIV risk and heterosexual transmission. *J. Natl. Med. Assoc.* 97 52S–59S. 16080458 PMC2640641

[B23] Mir D. Jung M. Delatorre E. Vidal N. Peeters M. Bello G. (2016). Phylodynamics of the major HIV-1 CRF02_AG African lineages and its global dissemination. *Infect. Genet. Evol.* 46 190–199. 10.1016/j.meegid.2016.05.017 27180893

[B24] Neogi U. Bontell I. Shet A. De Costa A. Gupta S. Diwan V. (2012). Molecular epidemiology of HIV-1 subtypes in india: origin and evolutionary history of the predominant subtype C. *PLoS One* 7:e39819. 10.1371/journal.pone.0039819 22768132 PMC3387228

[B25] Pang W. Zhang C. Duo L. Zhou Y.-H. Yao Z.-H. Liu F.-L. (2012). Extensive and complex HIV-1 recombination between B’, C and CRF01_AE among IDUs in south-east Asia. *AIDS* 26 1121–1129. 10.1097/QAD.0b013e3283522c97 22333750

[B26] Panichsillapakit T. Smith D. M. Wertheim J. O. Richman D. D. Little S. J. Mehta S. R. (2016). Prevalence of Transmitted HIV Drug Resistance Among Recently Infected Persons in San Diego, CA 1996–2013. *JAIDS J. Acquir. Immune Defic. Syndr.* 71 228–236. 10.1097/QAI.0000000000000831 26413846 PMC4712087

[B27] R Core Team (2013). *R: A Language and Environment for Statistical Computing.* Vienna: R Foundation for Statistical Computing.

[B28] Ragonnet-Cronin M. Hodcroft E. Hué S. Fearnhill E. Delpech V. Brown A. J. L. (2013). Automated analysis of phylogenetic clusters. *BMC Bioinformatics* 14:317. 10.1186/1471-2105-14-317 24191891 PMC4228337

[B29] Rambaut A. (2009). *FigTree v1.4: Tree Figure Drawing Tool.* Available at: http://tree.bio.ed.ac.uk/software/figtree/

[B30] Rambaut A. Lam T. T. Max Carvalho L. Pybus O. G. (2016). Exploring the temporal structure of heterochronous sequences using TempEst (formerly Path-O-Gen). *Virus Evol.* 2:vew007. 10.1093/ve/vew007 27774300 PMC4989882

[B31] Rose R. Lamers S. L. Dollar J. J. Grabowski M. K. Hodcroft E. B. Ragonnet-Cronin M. (2017). Identifying transmission clusters with cluster picker and HIV-TRACE. *AIDS Res. Hum. Retroviruses* 33 211–218. 10.1089/aid.2016.0205 27824249 PMC5333565

[B32] San Mateo County Department of Public Health (2017). *San Mateo County Sexually Transmitted Disease and HIV-AIDS Surveillance Annual Report.* San Francisco, CA: San Mateo County Department of Public Health.

[B33] San Mateo County Sexually Transmitted Disease and HIV-AIDS Surveillance Annual Report (2015). Available from https://www.smchealth.org/sites/main/files/file-attachments/2015_stdhiv_ar_final_12_28_16.pdf.

[B34] Sanchez M. A. Lemp G. F. Magis-Rodríguez C. Bravo-García E. Carter S. Ruiz J. D. (2004). The epidemiology of HIV among Mexican migrants and recent immigrants in California and Mexico. *J. Acquir. Immune Defic. Syndr.* 37(Suppl. 4), S204–S214. 10.1097/01.qai.0000141253.54217.24 15722863

[B35] Smith R. J. Okano J. T. Kahn J. S. Bodine E. N. Blower S. (2010). Evolutionary dynamics of complex networks of hiv drug-resistant strains: the case of San Francisco. *Science* 327 697–701. 10.1126/science.1180556 20075214

[B36] Stamatakis A. (2014). RAxML version 8: a tool for phylogenetic analysis and post-analysis of large phylogenies. *Bioinformatics* 30 1312–1313. 10.1093/bioinformatics/btu033 24451623 PMC3998144

[B37] Suchard M. A. Lemey P. Baele G. Ayres D. L. Drummond A. J. Rambaut A. (2018). Bayesian phylogenetic and phylodynamic data integration using BEAST 1.10. *Virus Evol.* 4 1–5. 10.1093/ve/vey016 29942656 PMC6007674

[B38] Tavaré S. (1986). Some probabilistic and statistical problems in the analysis of DNA sequences. *Lect. Math. Life Sci.* 17 57–86.

[B39] Thorne C. Ferencic N. Malyuta R. Mimica J. Niemiec T. (2010). Central Asia: hotspot in the worldwide HIV epidemic. *Lancet Infect. Dis.* 10 479–488. 10.1016/S1473-3099(10)70118-3 20610330

[B40] Wensing A. M. Calvez V. Günthard H. F. Johnson V. A. Paredes R. Pillay D. (2017). 2017 update of the drug resistance mutations in HIV-1. *Top. Antivir. Med.* 24 132–133. 28208121 PMC5677049

[B41] Wertheim J. O. Leigh Brown A. J. Hepler N. L. Mehta S. R. Richman D. D. Smith D. M. (2014). The global transmission network of HIV-1. *J. Infect. Dis.* 209 304–313. 10.1093/infdis/jit524 24151309 PMC3873788

[B42] World Health Organization. (2012). *WHO, UNODC, UNAIDS Technical Guide: For Countries to Set Targets for Universal Access to HIV Prevention, Treatment and Care for Injecting Drug Users 2012 Revision.* Geneva: World Health Organization.

