# Supplementary material for: Combining Phylogenetic and Network Approaches to Identify HIV-1 Transmission Links in San Mateo County, California
Source: Front Microbiol. 2018 Dec 6;9:2799. doi: 10.3389/fmicb.2018.02799 (PMC6292275; doi:10.3389/fmicb.2018.02799)
Supplement: Supplementary file 1 [file Table_1.DOCX]

**Supplementary Material:**

**Table S1.** Clustering results in Cluster Picker and HIV-TRACE for 316 HIV *pol* sequences from San Mateo County, California.

| **Cluster** | **Clustering Analysis** | | | |
| --- | --- | --- | --- | --- |
|  | **ClusterPicker** | **TBE Support** | **HIV-TRACE** | **Genetic Distance** |
|  | **Sequence ID** |  | **Sequence ID** |  |
| **1** | 14616 | 0.951 | 14616 | 0.03435114503816794 |
|  | 35580 |  | 35580 |  |
|  |  |  |  |  |
| **2** | 9894 | 1.0 | 9894 | 0.035305343511450385 |
|  | 21031 |  | 21031 |  |
|  |  |  |  |  |
| **3** | 1121 | 0.974265 | - | - |
|  | 1254 |  | 1254 |  |
|  | 1285 |  | 1285 |  |
|  | - |  | 1343 |  |
|  | 1344 |  | 1344 |  |
|  | - |  | 1422 |  |
|  | 1437 |  | - |  |
|  | 1451 |  | - |  |
|  | 1505 |  | 1505 |  |
|  | 1576 |  | 1576 |  |
|  | 3911 |  | - |  |
|  | - |  | 4015 |  |
|  | 4016 |  | - |  |
|  | 4055 |  | - |  |
|  | - |  | 4370 |  |
|  | - |  | 4381 |  |
|  | 4746 |  | - |  |
|  | 5101 |  | - |  |
|  | 5258 |  | 5258 |  |
|  | 5343 |  | 5343 |  |
|  | 5977 |  | 5977 |  |
|  | 5984 |  | - |  |
|  | - |  | 6351 |  |
|  | - |  | 6427 |  |
|  | 6460 |  | - |  |
|  | 6587 |  | - |  |
|  | 6811 |  | 6811 |  |
|  | - |  | 7072 |  |
|  | 7124 |  | - |  |
|  | 7864 |  | 7864 |  |
|  | 7967 |  | - |  |
|  | 8367 |  | 8367 |  |
|  | 8368 |  | 8368 |  |
|  | 8405 |  | 8405 |  |
|  | - |  | 9758 |  |
|  | 9780 |  | 9780 |  |
|  | 9797 |  | 9797 |  |
|  | - |  | 9858 |  |
|  | - |  | 9950 |  |
|  | - |  | 14258 |  |
|  | 14537 |  | - |  |
|  | - |  | 14554 |  |
|  | 14559 |  | 14559 |  |
|  | - |  | 14623 |  |
|  | 16161 |  | 16161 |  |
|  | - |  | 16331 |  |
|  | - |  | 16491 |  |
|  | 16547 |  | 16547 |  |
|  | 17701 |  | 17701 |  |
|  | 18323 |  | 18323 |  |
|  | 21093 |  | - |  |
|  | 21094 |  | 21094 |  |
|  | 21095 |  | 21095 |  |
|  | - |  | 21096 |  |
|  | 22139 |  | 22139 |  |
|  | 24978 |  | - |  |
|  | 25028 |  | - |  |
|  | 25581 |  | 25581 |  |
|  | 25667 |  | - |  |
|  | 26377 |  | 26377 |  |
|  | - |  | 27894 |  |
|  | 30181 |  | - |  |
|  | 35493 |  | - |  |
|  | - |  | 37875 |  |
|  | - |  | 39178 |  |
|  | 39202 |  | - |  |
|  | 39272 |  | - |  |
|  | 39280 |  | 39280 |  |
|  | 42056 |  | 42056 |  |
|  | 56109 |  | - |  |
|  |  |  |  |  |
| **4** | 21023 | 1.0 | 21023 | 0.02385496183206107 |
|  | 24956 |  | 24956 |  |
|  |  |  |  |  |
| **5** | 4570 | 0.975 | - | 0.03148854961832061 |
|  | 9825 |  | - |  |
|  |  |  |  |  |
| **6** | 3953 | 0.972 | 3953 | 0.020038167938931296 |
|  | 4007 |  | 4007 |  |
|  |  |  |  |  |
| **7** | 42129 | 1.0 | - | 0.03721374045801527 |
|  | 42672 |  | - |  |
|  |  |  |  |  |
| **8** | 4308 | 0.999 | 4308 | 0.04961832061068702 |
|  | 26421 |  | 26421 |  |
|  |  |  |  |  |
| **9** | 9758 | 0.997 | - | 0.042938931297709926 |
|  | 27894 |  | - |  |
|  |  |  |  |  |
| **10** | - | - | 9816 | 0.0335249042 |
|  | - |  | 37877 |  |
|  |  |  |  |  |
| **11** | - | - | 42129 | 0.0373563218 |
|  | - |  | 42672 |  |

Green cells denote a match of transmission links between two methods. Red cells denote a non-match between methodologies.
